# Supplementary material for: Healthcare outcomes and special education eligibility in children with congenital CMV
Source: PLoS One. 2024 Dec 19;19(12):e0313163. doi: 10.1371/journal.pone.0313163 (PMC11658512; doi:10.1371/journal.pone.0313163)
Supplement: S1 Table — Listing of ICD-10-CM codes that have been grouped as guided by the SNOMED-CT. (PDF) [file pone.0313163.s001.pdf]

Supplementary Table 1:

SNOMED-CT® ICD-10 Code Groupings
Below is the list of ICD-10-CM codes that have been grouped as guided by the SNOMED-CT.

Hearing Problem
H905, H9325

Vision Problem
H3320, H4720, H47619, H538

Hearing Devices
Z9621, Z974, F06ZBLZ

Central Nervous System Problem
G800, G801, G803, G804, G809

Neuro-muscular Problem
F82, M624, P941, R29898, R6250

Developmental delays and disabilities
F70, F71, F72, F73, F78, F79, F800, F801, F802, F804, F808, F809, F819, F840, F848, F88, F89, F06ZBZZZ, R4183, R620

Daily Living, Functioning, and Assistive Aids
R262, R278, Z740, Z7409, Z741, Z7689, Z931, Z993, F07M3ZZ, Z9989
