# Supplementary material for: Healthcare outcomes and special education eligibility in children with congenital CMV
Source: PLoS One. 2024 Dec 19;19(12):e0313163. doi: 10.1371/journal.pone.0313163 (PMC11658512; doi:10.1371/journal.pone.0313163)
Supplement: Attachment — Submitted filename: Response to Reviewers - PLOS for Resubmit.docx [file pone.0313163.s002.docx]

**Response to Journal Requirements**

**Requirement 1:**

We have reformatted the paper in accordance with the materials provided on the PLoS website for naming and style.

**Requirement 2:**

We have removed funding information from the manuscript and enter a corrected version of this on the online submission form. We have left the competing interest and funding statements at the top of the manuscript.

**Requirement 3:**

We apologize for the confusion. This work was not grant funded. It was supported through a contractual agreement between Baylor College of Medicine and Merck. We have clarified this in the Funding statement and updated the ‘Funding Information’ and ‘Financial Disclosure’ statements so that they are in agreement and read as:

Funding for this research was provided by a contractual agreement between Baylor College of Medicine and Merck Sharp & Dohme LLC, a subsidiary of Merck & Co., Inc., Rahway, NJ, U.S.A.

**Requirement 4:**

We have clarified the role of the sponsor in the manuscript (as two authors are employees of the funder). The role of the funder is explained in both the “Role of Funder/Sponsor” and “Contributors’ Statement” which have been amended, as requested, in the requirements document sections and the cover letter to state:

Drs. Goodman and Wang are employees of the funder/sponsor and helped conceptualize the study, provided guidance on analytic approach, reviewed and interpreted the results, and edited and reviewed the manuscript. The funder provided support in the form of salaries for all authors (RHR, EG, WW, JM, GJD) but did not have any additional role in the study design, data collection and analysis, decision to publish, or preparation of the manuscript. The specific roles of these authors are articulated in the ‘author contributions’ section.

**Requirement 5:**

We have updated the funding statement in the manuscript and cover letter, as well as in the online submission page as requested. They now read:

**Funding Statement:** Drs. Goodman and Wang are employees of the funder/sponsor and helped conceptualize the study, provided guidance on analytic approach, reviewed and interpreted the results, and edited and reviewed the manuscript. The funder provided support in the form of salaries for all authors (RHR,EG, WW, JM, GJD) but did not have any additional role in the study design, data collection and analysis, decision to publish, or preparation of the manuscript. The specific roles of these authors are articulated in the ‘author contributions’ section.

**Competing Interests:** Drs. Goodman and Wang are employees of Merck Sharp & Dohme LLC, a subsidiary of Merck & Co., Inc., Rahway, NJ, USA and may hold stock in Merck & Co., Inc., Rahway, NJ, USA. Drs. Rochat, Miller, and Demmler-Harrison received research support from Merck & Co, Inc. The role of the funder does not alter our adherence to PLOS ONE policies on sharing data and materials as this is prescribed within and by the Institutional Review Board of Baylor College of Medicine and Affiliated Institutions: (H-46267) Health care costs associated with congenital cytomegalovirus (CMV) infection and disease. Drs. Miller and Demmler-Harrison have also received research support from Microgen Laboratories.

**Requirement 6:**

In regards to the availability of data for this project, owing to the vulnerable nature of the patients included in this study (children, cognitive impairment), sharing of a full de-identified data set for the purposes of publication is limited by the Institutional Review Board of Baylor College of Medicine and Affiliated Institutions (Protocol: H-46267) which states “when analyzed and presented or published, will be presented in aggregate data only”. As such, data within this manuscript can be made available upon request, and the decision to make available is not restricted by the funder/sponsor. Contact information for the Baylor College of Medicine Institutional Review Board of Baylor College of Medicine and Affiliated Institutions is as follows: irb@bcm.edu, phone 713-798-6970.

**Requirement 7:**

We have addended the Methods(Data Acquisition) section to include a statement to this effect which reads:

Data collection and analysis for this project was approved by the Institutional Review Board of Baylor College of Medicine and Affiliated Institutions approved (Protocol: H-46267) “Health care costs associated with congenital cytomegalovirus (CMV) infection and disease.” As this was a retrospective chart review, consent was waived by the Baylor College of Medicine and Affiliated Institutions Institutional Review Board.

**Requirement 8:**

Our references are complete and correct and there have been no redactions.

**Response to Reviewers**

**Reviewer 1:**

**This is an important paper demonstrating the global impact of cCMV whether or not asymptomatic at birth on the long term outcomes of children. The breakdown of specific conditions that qualify for IDEA is interesting and helpful to explore the variety of needs these children can have. There are a few clarifying questions that arise from the paper.**

Thank you for these comments. We are glad to see that the reviewer agrees with an approach looking at the real-world utilization of these children as a marker of the needs of these children, specifically those classified as asymptomatic at birth. We hope our responses to your questions below are of help and we will make sure that they are addressed within the manuscript as well.

**1. How did you identify the AcCMV cohort in the clinical arm since they were not part of the large prospective Houston CMV study?**

Children with AcCMV in the clinical cohort were classified, similarly to those in the historical cohort, as those who did not have any of the following (lines 153-157): purpura/petechiae, jaundice, hepatosplenomegaly, microcephaly, unexplained neurological abnormality, elevated liver enzymes, hyperbilirubinema, hemolytic anemia or thrombocytopenia. Since we were not routinely screening for CMV in the clinical cohort, these were patients who came into our care through referral for any of a variety of reasons, either failed hearing screening at birth, or screening through parental concern. We have amended the manuscript in the methods section to highlight this fact.

**2. Were ICD9 and ICD 10 codes used or were the ICD9 codes from the historical cohort reclassified into ICD10? It seems you mainly used the ICD10 codes to translate to the IDEA qualifying conditions.**

For the historical cohort, the codes we used were manually abstracted from historical records (typically upon review of paper charts) into the ICD10 ontology. As such, there was no need to translate from ICD9 to ICD10. For the clinical cohort, all codes were extracted from the electronic medical record. In accordance with regulations from the Centers for Medicare & Medicaid Services, all codes within our system were migrated from ICD9 to ICD10 years prior to our extraction. In our system, this migration was retrospective, and so when extracting these codes in 2021, the ICD codes had already been translated into the ICD10 ontology. We have modified the text of the manuscript in the methods section to clarify that all codes were ICD10 when manually abstracted at time of extraction.

**3. Why do you suppose there was a much higher percent in the AcCMV who met IDEA criteria in the clinical cohort? Could that be related to the exclusion criteria mentioned in the historical group?**

The classification of AcCMV and ScCMV was similar across the two cohorts, with AcCMV being those infants with positive CMV testing who did not have any of the following at birth: purpura/petechiae, jaundice, hepatosplenomegaly, microcephaly, unexplained neurological abnormality, elevated liver enzymes, hyperbilirubinema, hemolytic anemia or thrombocytopenia. Since AcCMV cases in the historical cohort were identified in through universal screening, it is possible that this approach was more sensitive in identifying patients than what was seen in the clinical cohort which represents a real-world view of referrals for this congenital infection. As we note in the limitations section, the clinical cohort is a reflection of referrals to center, and so there is the potential for referral bias in this group. This being said, we feel that the higher percentage in the AcCMV who met criteria in the clinical cohort is due to the real-world evaluation of these children. What that means is that in comparing the historical to clinical cohort, in the historical cohort these codes would have been abstracted upon review of documentation from that study, where as the codes for the clinical cohort came from tens of thousands of provider encounters across dozens of specialties. This is not to say that the documentation in the historical cohort was not as complete, merely that the historical documentation was from a single provider throughout the course of that study, versus a reflection of the evaluations of thousands of providers in the contemporary clinical cohort. We have added a section in the discussion to address this.

**Reviewer 2:**

**This study tried to find need for special education in children suffering from congenital cytomegalovirus infection, a nice study explored underaddressed issues. hence I have few observation.**

Thank you for these comments. We hope our responses to your questions below are of help and thank you for helping improve the content and readability of the paper.

**1. Title need to be self explanatory for better understanding of readers**

We have changed the title to make it more self explanatory. The current title is now:

Healthcare Outcomes and Special Education Eligibility in Children with Congenital CMV

**2.Limitation of study need to be brief.**

We have shortened this section as requested.
